# Prevalence of hypokalemia in older persons: results from the PolSenior national survey

**DOI:** 10.1007/s41999-021-00484-6

**Published:** 2021-04-08

**Authors:** Marcin Adamczak, Jerzy Chudek, Jan Zejda, Magdalena Bartmańska, Tomasz Grodzicki, Tomasz Zdrojewski, Andrzej Wiecek

**Affiliations:** 1 Department of Nephrology, Transplantation and Internal Medicine, Medical University of Silesia in Katowice, Francuska Str. 20/24, 40-027 Katowice, Poland; 2 Department of Internal Diseases and Oncological Chemotherapy, Medical University of Silesia in Katowice, Katowice, Poland; 3 Department of Epidemiology, Medical University of Silesia in Katowice, Katowice, Poland; 4 Department of Internal Medicine and Gerontology, Jagiellonian University Medical College, Cracow, Poland; 5 Department of Hypertension and Diabetology, Medical University of Gdansk, Gdansk, Poland

**Keywords:** Diuretic therapy, Older persons, Hypertension, Hypokalemia, Prevalence

## Abstract

**Purpose:**

Hypokalemia is one of the most common electrolyte disturbances in clinical practice. There are only a few epidemiological studies analyzing the occurrence of hypokalemia in older persons. The aim of the study was to determine the prevalence of hypokalemia in the Polish older population.

**Methods:**

Serum potassium concentration was estimated in 4654 participants (2270 females and 2384 males, mean age 76.5 [11.0] years), who participated in the PolSenior study. Hypokalemia was defined as serum potassium concentration below 3.5 mmol/L. Hypokalemia was found in 39 participants (0.84%) and was significantly more frequent among females (28 females = 1.23% and 11 males = 0.46%; *p = *0.003). The prevalence of hypokalemia was not related to age. Among 3303 participants suffering from arterial hypertension, 1093 were treated with potassium-losing diuretics.

**Results:**

Hypokalemia was significantly more frequent among hypertensive than normotensive older participants (1.06 vs. 0.30% respectively; *p = *0.007) and among hypertensive participants treated with potassium losing diuretics than ones untreated with these drugs (1.96 vs. 0.46% respectively; *p* < 0.001). In hypertensive participants, the prevalence of hypokalemia did not depend significantly on oral supplementation of potassium (1.92 and 0.98% respectively, NS). None of 81 participants using laxative agents presented hypokalemia.

**Conclusions:**

This study demonstrates that: older age seems not to appear to be a significant risk factor of hypokalemia. Hypokalemia is more often found in the older hypertensive patients treated with potassium losing diuretics, and prevention of diuretic-induced hypokalemia with oral supplementation of potassium seems to be insufficient.

## Key summary points

**Aim:**

To examine what is the prevalence of hypokalemia in the older population.

**Findings:**

The prevalence of hypokalemia was similar in older persons and younger ones. Hypokalemia is more often found in patients with arterial hypertension treated with diuretics. Oral supplementation of potassium in these patients did not prevent hypokalemia.

**Message:**

In older persons, diuretic treatment is the most important cause of hypokalemia. Prevention of hypokalemia with the use of oral potassium supplementation is in this population insufficient.

## Introduction

Hypokalemia is one of the most common electrolyte disturbances seen in clinical practice [1]. Although thresholds for the definition of hypokalemia vary slightly, a widely quoted lower limit for a normal serum potassium concentration is 3.5 mmol/L [1].

Hypokalemia can be a life-threatening condition, due to its association with cardiac arrhythmias [2], stroke risk, and sudden death [3, 4]. Patients diagnosed with coronary artery disease (CAD) are especially at high risk of hypokalemia-induced arrhythmias. The risk related to hypokalemia in patients suffering from CAD is well documented [5, 6]. In one study [7], the mortality rate of hospitalized hypokalemic patients was tenfold higher than that of the general hospitalized population. It has also been shown that hypokalemia is more frequently associated with worsening outcomes in patients attending emergency departments even in an initially low-risk population [8]. Therefore, hypokalemia seems to be of particular importance in older persons due to the high prevalence of CAD.

In older persons due to multimorbidity polypharmacy is common, therefore the risk of electrolyte disturbances caused by drugs is greater than in the general population [9].

Correction of hypokalemia is simple and inexpensive, consisting of potassium supplementation or the initiation of potassium-sparing medications. There is a general agreement that immediate potassium supplementation should be given at serum levels of less than 3.0 mmol/L, because of the increased risk of life-threatening arrhythmias below this level [10].

As the correction of hypokalemia is not difficult, a more important issue seems to be the identification of patients who are especially prone to hypokalemia development. Until now there are only a few population-based studies on the occurrence of hypokalemia in older persons [11, 12]. Therefore the aim of the study was to analyze the frequency of hypokalemia in the Polish older persons recruited from the general population, participating in the PolSenior project.

## Patients and methods

The precise study design of the PolSenior project was described by Bledowski et al. [13]. The study protocol was approved by the Bioethics Committee of the Medical University of Silesia in Katowice, Poland (KNW-6501-38/I/08). The approval concerned among others informed consent form. Briefly, the study group included 5695 respondents (2899 males and 2796 females) split into six equally sized age groups of older persons (65–69 years, 70–74 years, 75–79 years, 80–84 years, 85–89 years and over 90 years old) and an internal reference group of participants aged 55–59 years. Participants were recruited using three stages stratified, proportional sampling. The first stage identified local administrative units, including urban, rural and urban–rural municipalities. During the second stage, streets in urban municipalities, and villages in rural municipalities were selected. The third stage enabled random recruitment of individuals in bundles and was executed using the national PESEL database (Universal Electronic System for Registration of the Population).

In all participants, the medical history (including the presence of hypertension, diabetes mellitus, CAD or heart failure) and data concerning medications (particularly treatment with diuretics) were collected.

Venous blood samples were collected using a vacuum tube system. Blood samples were delivered within 2 h. to local laboratories, where serum samples were separated, frozen, and delivered to the Central Laboratory.

Serum potassium and creatinine concentrations were measured in 4654 participants (2270 females and 2384 males); 625 participants aged 55–59, 683 participants aged 65–69 years, 778 participants 70–74 years, 698 participants aged 75–79 years and 626 participants aged 80–84 years and 562 participants 90 or more years old. Serum potassium concentration was measured with the use of ion-selective electrode technique and serum creatinine concentration with the routine laboratory method. The estimated glomerular filtration rate (eGFR) was calculated according to the CKD-EPI formula [14].

### Data analysis

Hypokalemia was defined as serum potassium concentration lower than 3.5 mmol/L, while severe hypokalemia was defined by values lower than 3.0 mmol/L.

Arterial hypertension was diagnosed when during each of two visits, the average (from two measurements) systolic blood pressure was at least 140 mmHg and/or average from two measurements of diastolic blood pressure was at least 90 mmHg or the participant was receiving antihypertensive medications. Obesity was defined as body mass index (BMI) ≥ 30 kg/m^2^. An epidemiological diagnosis of diabetes was established based on medical history and medications. Respondents were considered as having heart failure when plasma NT-proBNP level exceeded 2000 pg/mL, while values below 400 pg/mL were scored as no heart failure [15].

### Statistical analysis

The distribution of quantitative variables was presented by arithmetic means and standard deviations (SD), and of qualitative variables by absolute and relative frequencies. Because of the abnormal distribution of kalemia (result of Shapiro–Wilk test) statistical significance of between-group differences was examined by means of the Kruskal–Wallis and Wilcoxon tests. Statistical significance of differences in the frequencies of hypokalemia was analyzed using chi-square test, chi-square test for trend or Fisher test, if number of observations was small. Correlation analyses were performed using the Spearman method. All statistical analyses were performed using SAS software version 9.1.3 (SAS Institute Inc., Cary, NC) and the statistical inference was based on the criterion *p* < 0.05.

## Results

In the entire group of males, serum potassium concentration was significantly higher than in females (4.55 [0.49] vs. 4.47 [0.48] mmol/L; *p* < 0.001). Hypokalemia was found in 39 participants (0.84%) and was significantly more frequent among females (28 females—1.23% and 11 males—0.46%; *p = *0.003). No case of severe hypokalemia was found. In the entire group of males eGFR was significantly higher than in females (72.5 [18.3] vs. 70.9 [18.6] mL/min/1.73m^2^ respectively; *p* < 0.001). eGFR was similar in participants with or without hypokalemia (71.9 [17.9] vs. 71.7 [18.5] mL/min/1.73m^2^ respectively).

### Serum potassium concentration, eGFR and hypokalemia in different age groups

**Fig. 1:**
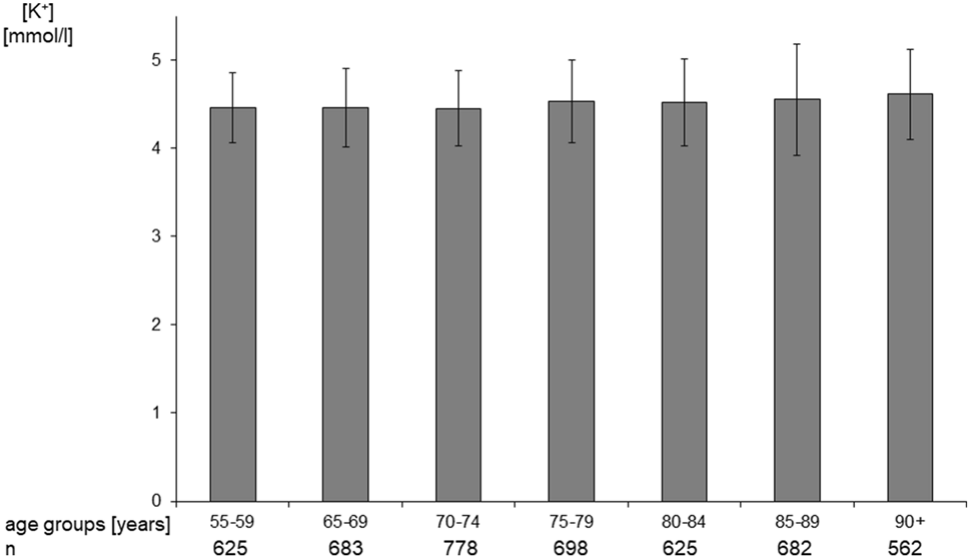
Serum potassium concentration in different age groups

As shown on Fig. 1 serum potassium concentration was increasing slightly, however significantly (*p* < 0.001) with age. It was significantly higher in participants 65 years or older than in participants aged 55–59 years (4.51 [0.50] vs. 4.46 [0.40] mmol/L respectively; *p* = 0.003). However, the frequency of hypokalemia was similar in the studied age groups (data not shown), and also in the entire group of 65 years old and from the group 55–59 years old (0.32 vs. 0.92%). Among all studied participants, 1188 of them were characterized with eGFR below 60 mL/min/1.73m^2^ (25.5% of entire study group). In the entire studied group a significant, negative correlation was found between serum potassium concentration and eGFR (*r* = − 0.207; *p* < 0.001). Moreover, serum potassium concentration was significantly higher in participants with eGFR < 60 mL/min/1.73 m^2^ than in participants with higher eGFR (4.64 [0.59] vs. 4.46 [0.44] mmol/L respectively; *p* < 0.001). However, the prevalence of hypokalemia was similar in both groups (0.76 and 0.89%, respectively).

### Serum potassium concentration and hypokalemia in hypertensive participants

**Fig. 2:**
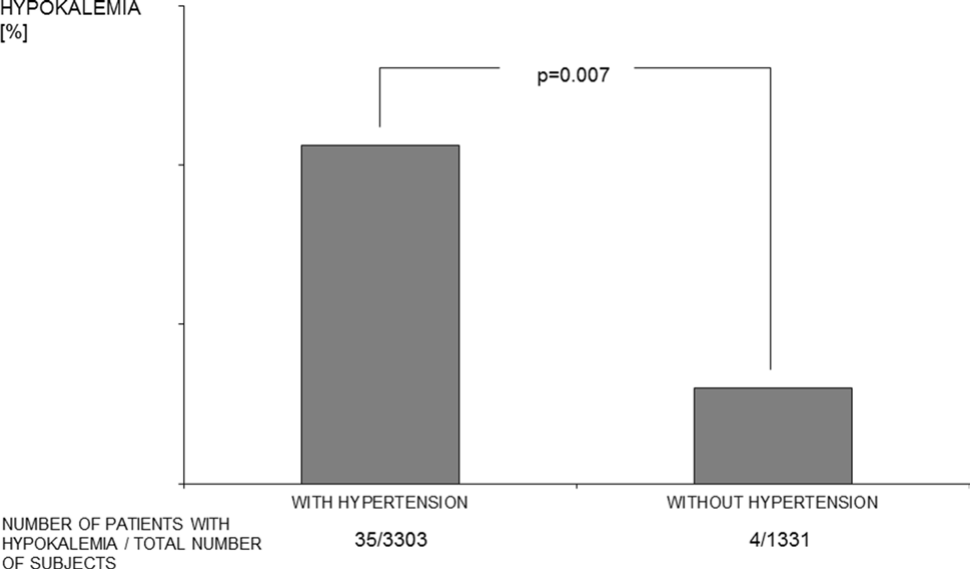
Frequency of hypokalemia in subjects with and without hypertension

From the entire study group, 3303 participants had arterial hypertension (71%). Serum potassium concentration was slightly, however significantly lower in hypertensive participants compared to normotensive ones (4.49 [0.46] vs. 4.56 [0.51] mmol/L respectively; *p* < 0.001)). Hypokalemia was significantly more frequently diagnosed among hypertensive than among normotensive participants (1.06 vs. 0.30% respectively; *p = *0.007) (Fig. 2).

### Serum potassium concentration and hypokalemia in obese participants

Obesity was found in 1429 participants (30.7% of the entire study group with serum potassium measurements). Both serum potassium concentration and frequency of hypokalemia were similar in obese and non-obese participants (4.50 [0.48] vs. 4.52 [0.49] mmol/L and 0.91 and 0.75%, respectively).

### Serum potassium concentration and hypokalemia in participants with diabetes mellitus

In the study group, 1011 participants were diagnosed for diabetes (21.7% of an entire study group with serum potassium measurements). Serum potassium concentration was slightly, not significantly higher in diabetic participants (4.54 [0.47] vs. 4.50 [0.56] mmol/L respectively; *p = *0.07). In contrast, hypokalemia was significantly more frequent among diabetic than nondiabetic participants (1.38 vs. 0.69% respectively; *p = *0.04).

### Serum potassium concentration and hypokalemia in participants with CAD

CAD was diagnosed in 613 participants (13.2% of entire study group with serum potassium measurements). Serum potassium concentration was slightly, however significantly higher in participants with than in those without CAD (4.57 [0.60] vs. 4.50 [0.47] mmol/L respectively; *p* < 0.01). The frequency of hypokalemia was similar in both groups (0.65 and 0.87%, respectively).

### Serum potassium concentration and hypokalemia in participants with heart failure

Diagnosis of heart failure was established in 454 participants (9.8% of entire study group with serum potassium measurements). Serum potassium concentration was slightly, however significantly higher in participants than in those without heart failure (4.61 [0.67] vs. 4.50 [0.46] mmol/L respectively, *p* = 0.004). The frequency of hypokalemia were similar in both groups (1.10 and 0.82%, respectively).

### Serum potassium concentration and hypokalemia in participants using laxative agents

Among the studied participants, 81 used chronically laxative agents (1.7% of entire study group). Both serum potassium concentration and frequency of hypokalemia were similar in participants using and not using laxative agents (4.45 [0.49] vs. 4.51 [0.49] mmol/L and 0 and 0.86%, respectively).

### Serum potassium concentration and hypokalemia in hypertensive participants treated with potassium losing diuretics

**Fig. 3:**
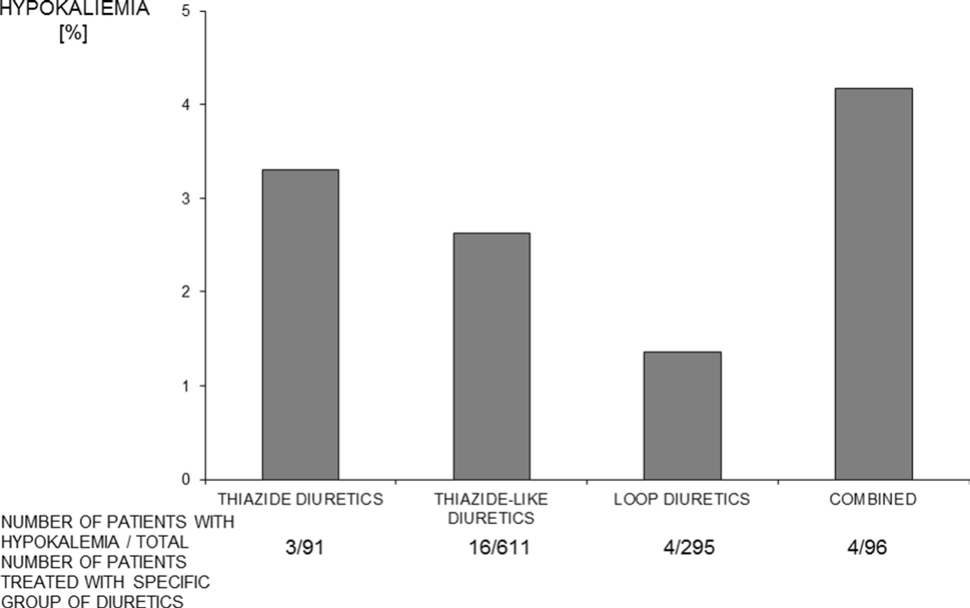
Frequency of hypokalemia in hypertensive patients treated with different potassium losing diuretics

In the subgroup of 3303 hypertensive participants (1619 males and 1684 females; mean age 75.9 [10.3] years), 1093 were treated with potassium losing diuretics (91 with thiazide diuretics, 611 with thiazide-like diuretics, 295 loop diuretics and 96 with a combination of amiloride and hydrochlorothiazide). Hypertensive patients treated with thiazide-like diuretics were characterized by significantly lower serum potassium concentration than not treated with these agents (Table 1). Hypertensive participants treated with potassium losing diuretics were characterized by a significantly higher frequency of hypokalemia than untreated hypertensive individuals (1.96 vs. 0.46% respectively; *p* < 0.001). The frequency of hypokalemia was significantly higher among hypertensive patients treated with thiazide-like diuretics and with a combination of amiloride and hydrochlorothiazide than among those not treated with these agents (Table 1, Fig. 3).

**Table 1:** Serum potassium concentration, number of cases, and frequency of hypokalemia in hypertensive patients treated and not treated with thiazide, thiazide-like, loop diuretics and combination of amiloride and hydrochlorothiazide

Class of diuretics		Patients	Serum potassium concentration	Cases of hypokalemia	
		*n*	mmol/L	*n*	%
Thiazide	Treated	91	4.53 (0.74)	3	3.30
	Untreated	3212	4.49 (0.47)	32	1.01
	*p*	ns		ns	
Thiazide-like	Treated	611	4.29 (0.46)	16	2.62
	Untreated	2652	4.53 (0.47)	19	0.72
	*p*	< 0.0001		< 0.0001	
Loop	Treated	295	4.67 (0.59)	4	1.36
	Untreated	3008	4.47(0.46)	31	1.04
	*p*	< 0.0001		ns	
Amiloride and hydrochloro-thiazide	Treated	96	4.46 (0.57)	4	4.17
	Untreated	3207	4.49 (0.47)	31	0.98
	*p*	ns		0.01	

*ns* statistically not significant

Among 1093 hypertensive participants treated with potassium losing diuretics, 313 (28.6%) received oral potassium supplementation (20.9% treated with thiazide diuretics, 14.1% with thiazide-like diuretics, 32.2% with loop diuretics and 16.7% with a combination of amiloride and hydrochlorothiazide). Serum potassium concentration and frequency of hypokalemia were similar in hypertensive participants treated with potassium losing diuretics receiving and not receiving potassium oral supplementation (4.47 [0.51] vs. 4.49 [0.48] mmol/L and 1.92 and 0.98%, respectively). Moreover, serum potassium concentration and frequency of hypokalemia were similar in hypertensive patients treated with different classes of diuretics and receiving or not receiving potassium oral supplementation. Precisely, 5.26% (1 of 19) patients treated with thiazide diuretics and receiving oral potassium supplementation had hypokalemia in comparison to 2.78% (2 of 72) of patients without potassium supplementation. Comparing the frequency of hypokalemia in patients treated with thiazide-like and receiving oral potassium with ones without supplementation was 4.65% (4 of 86) and 2.25% (12 of 525) respectively. None of hypertensive patients receiving oral potassium supplementation and loop diuretics had hypokalemia and 2% (4 of 200) not receiving ones. In a group of patients treated with amiloride and hydrochlorothiazide combined with oral potassium supplementation 6.25% (1 of 16) suffers from hypokalemia, while the prevalence of hypokalemia in patients not receiving supplementation was 3.75% (3 of 80).

### Serum potassium concentration and hypokalemia in hypertensive participants treated with spironolactone

Among 3303 hypertensive participants 277 were treated with a potassium-sparing diuretic—spironolactone (8.4% of entire hypertensive studied participants). Hypertensive patients treated with spironolactone were characterized by significantly higher serum potassium concentration than those not treated with this potassium-sparing diuretic (4.76 [0.50] vs. 4.46 [0.47] mmol/L respectively; *p* < 0.001). There was no case of hypokalemia in hypertensive participants treated with spironolactone.

## Discussion

The main finding of our analysis is the fact, that the occurrence of hypokalemia in the older persons is similar to the figures obtained in the middle-age group. Therefore the aging per se seems not to be a risk factor of hypokalemia. In our study, we did not confirm the results of smaller studies which showed an increased frequency of hypokalemia in the older persons [11, 12]. In the present study, mean serum potassium concentration increased slightly, however significantly with age. A significant, negative correlation between serum potassium concentration and eGFR may suggest that increase in serum potassium could be due to an age-related decline of kidney function.

In this large-scale, population-based study, hypertensive persons were identified as the group with the increased risk of hypokalemia. The increased risk of hypokalemia in these patients was related to the treatment with diuretics (especially thiazide-like diuretics and a combination of amiloride and hydrochlorothiazide).

In the PolSenior study, the frequency of hypokalemia in hypertensive individuals treated with potassium-losing diuretics was 1.96%. The prevalence of diuretic-induced hypokalemia varies from one study to another. Earlier studies have found a higher frequency of hypokalemia in general and in older persons treated with diuretics [16–20]. One report showed that diuretic-induced hypokalemia was the most prevalent adverse drug reaction in the older hospitalized population both during hospitalization and at the time of admission [16]. In another study, 33% of older patients taking furosemide were hypokalemic [17]. Another observational study found hypokalemia in 8.5% of patients treated with low doses of thiazide diuretic [18]. In the SHEP study, 7.5% of patients receiving chlorthalidone developed hypokalemia after one year of the treatment [19]. The differences in frequency of diuretic-induced hypokalemia may be due to the heterogeneity of diuretic doses among these studies i.e. higher in earlier studies and lower (due to current recommendations) in more recent ones.

In PolSenior study, hypokalemia tended to be more frequent in patients treated with thiazide-like diuretics and a combination of amiloride and hydrochlorothiazide than in the patients treated with the loop diuretics. Therefore the presented results are in line with a view that loop diuretics seem to produce less average fall of potassium concentration compared with thiazides [21].

Diuretic-induced hypokalemia may lead to glucose intolerance [22]. It has been also implicated as a cause of new-onset diabetes mellitus. The exact mechanism by which hypokalemia may cause glucose intolerance is still unclear. It seems to be related to the inhibition of insulin secretion by the beta cells of the pancreas or decreasing plasma adiponectin concentration [22, 23]. Lai et al. showed that not only hypokalemia but low-normal serum potassium is associated with a significantly higher risk of cardiovascular and all-cause mortality in elderly outpatient care [24].

We have found that the frequency of hypokalemia was similar in hypertensive participants treated with potassium losing diuretics receiving and not receiving potassium oral supplementation. Such a finding suggests that the prevention of diuretic-induced hypokalemia with the application of potassium oral supplementation is ineffective. However, we did not have access to data concerning individual doses of supplemented potassium and a more specific discussion of that issue is not possible. Moreover, it is well known that oral potassium supplementation is not well tolerated in some of the patients.

There was no hypokalemia in hypertensive participants treated with spironolactone. That finding seems to confirm that the use of spironolactone, in contrast to oral potassium supplementation, is an effective method of prevention of hypokalemia.

It is well known that laxative abuse may cause excessive potassium loss in the stool and lead to hypokalemia [25, 26] and in all cases of hypokalemia, laxative abuse must be addressed while taking the patient history. In the presented study, both serum potassium concentration and frequency of hypokalemia was similar in participants using and not using laxative agents. Therefore, the risk of laxative-induced hypokalemia in the general older population is low and should not be overestimated.

In the PolSenior study, hypokalemia was significantly more frequent in females (1.23%) than in males (0.46%) and that gender-related gradient was found by other authors [27, 28]. The increased risk of hypokalemia in women might be related to their lower muscle mass and a smaller pool of exchangeable potassium [1, 29].

It cannot be forgotten that hypokalemia in older population, might be caused by redistribution of potassium from the intravascular to the extravascular compartment induced by drugs (mostly by insulin in diabetic patients). Hypokalemia may be also one of the side effects of, so common in older persons, polypharmacy.

Although it has been shown that the systemic renin–angiotensin system (RAS) is suppressed with age [30], activation of tissue RAS may play an important role in the pathogenesis of hypokalemia in older patients.

The strength of the PolSenior study is a design ensuring that the studied population is representative for the general Polish older population. Serum potassium concentration was also measured centrally in the accredited laboratory thus excluding between-observer variability.

The major limitation of our study stems from its cross-sectional design. Such a protocol hampers more specific insight into biological mechanisms involved in determinants of hypokalemia in older persons. Moreover, a number of data were derived from the questionnaire. Both factors might have affected the results. However, the principal goal of the PolSenior study was observational and the findings showed some associations that are biologically plausible.

Another potential limitation is the fact that the serum potassium concentration was not measured immediately after blood collection (which was infeasible in such, large-scale, population-based study). Blood samples were collected at the participant’s residence and then delivered within 2 h. to local laboratories, where serum samples were separated, frozen and subsequently transferred to the central laboratory. However, the samples with visible hemolysis were excluded from further investigations. However, we are not able to prove that in some samples slight hemolysis did not occur during preanalytical preparations. This could increase the results of serum potassium concentration, reducing the frequency of diagnosed hypokalemia.

In summary, we have found in our large scale, population-based study PolSenior that serum potassium concentration is slightly increasing with age in the apparently healthy older persons and aging seems not to be a significant risk factor of hypokalemia. Hypokalemia is more often found in older hypertensive patients, particularly in those treated with a potassium-losing diuretic. The prevention of diuretic-induced hypokalemia with the use of potassium oral supplementation seems to be ineffective. The risk of laxative-induced hypokalemia in the general older population is low and should not be overestimated.

## References

[1] Unwin RJ, Luft FC, Shirley DG (2011). Pathophysiology and management of hypokalemia: a clinical perspective. Nat Rev Nephrol.

[2] Danielsson B, Collin J, Nyman A (2020). Drug use and torsades de pointes cardiac arrhythmias in Sweden: a nationwide register-based cohort study. BMJ Open.

[3] Mattsson N, Nielsen OW, Johnson L (2018). Prognostic impact of mild hypokalemia in terms of death and stroke in the general population-a prospective population study. Am J Med.

[4] Palmer BF, Clegg DJ (2019). Physiology and pathophysiology of potassium homeostasis: core curriculum. Am J Kidney Dis.

[5] Alfonzo AV, Isles C, Geddes C, Deighan C (2006). Potassium disorders—clinical spectrum and emergency management. Resuscitation.

[6] Osadchii OE (2010). Mechanisms of hypokalemia-induced ventricular arrhythmogenicity. Fundam Clin Pharmacol.

[7] Paltiel O, Salakhov E, Ronen I (2001). Management of severe hypokalemia in hospitalized patients: a study of quality of care based on computerized databases. Arch Intern Med.

[8] Vuillaume LA, Ferreira JP, Asseray N (2020). Hypokalemia is frequent and has prognostic implications in stable patients attending the emergency department. PLoS ONE.

[9] Bień B, Bień-Barkowska A (2028). Prescribing or deprescribing in older persons: what are the real-life concerns in geriatric practice?. Pol Arch Intern Med.

[10] Newmark SR, Dluhy RG (1975). Hyperkalemia and hypokalemia. JAMA.

[11] Touitou Y, Godard JP, Ferment O (1987). Prevalence of magnesium and potassium deficiencies in the elderly. Clin Chem.

[12] Passare G, Viitanen M, Törring O (2004). Sodium and potassium disturbances in the elderly: prevalence and association with drug use. Clin Drug Investig.

[13] Bledowski P, Mossakowska M, Chudek J (2011). Medical, psychological and socioeconomic aspects of aging in Poland: assumptions and objectives of the PolSenior project. Exp Gerontol.

[14] Levey AS, Stevens LA, Schmid CH (2009). CKD-EPI (Chronic Kidney Disease Epidemiology Collaboration): a new equation to estimate glomerular filtration rate. Ann Intern Med.

[15] Dickstein K, Cohen-Solal A, Filippatos G et al (2008) ESC Committee for Practice Guidelines (CPG): ESC guidelines for the diagnosis and treatment of acute and chronic heart failure 2008: the Task Force for the diagnosis and treatment of acute and chronic heart failure 2008 of the European Society of Cardiology. Developed in collaboration with the Heart Failure Association of the ESC (HFA) and endorsed by the European Society of Intensive Care Medicine (ESICM). Eur J Heart Fail 10: 933–989 10.1016/j.ejheart.2008.08.005 18826876

[16] Passarelli MC, Jacob-Filho W, Figueras A (2005). Adverse drug reactions in an elderly hospitalised population: inappropriate prescription is a leading cause. Drugs Aging.

[17] Hamdy RC, Tovey J, Perera N (1980). Hypokalaemia and diuretics. BMJ.

[18] Clayton JA, Rodgers S, Blakey J (2006). Thiazide diuretic prescription and electrolyte abnormalities in primary care. Br J Clin Pharmacol.

[19] Franse LV, Pahor M, Di Bari M (2000). Hypokalemia associated with diuretic use and cardiovascular events in the Systolic Hypertension in the Elderly Program. Hypertension.

[20] Valentova M, Patel S, Lam PH (2020). Hypokalaemia and outcomes in older patients hospitalized for heart failure. ESC Heart Fail.

[21] Salem CB, Hmouda H, Bouraoui K (2009). Drug-induced hypokalaemia. Curr Drug Saf.

[22] Lithell HO (1991). Effect of antihypertensive drugs on insulin, glucose and lipid metabolism. Diabetes Care.

[23] Piecha G, Adamczak M, Chudek J, Więcek A (2007). Indapamide decreases plasma adiponectin concentration in patients with essential hypertension. Kidney Blood Press Res.

[24] Lai YH, Leu HB, Yeh WT (2015). Low-normal serum potassium is associated with an increased risk of cardiovascular and all-cause death in community-based elderly. J Formos Med Assoc.

[25] Kallmeyer JC, Macleod IN, Bhagwan B (1994). Marked hypokalaemic rhabdomyolysis due to purgative abuse. S Afr Med J.

[26] Chin RL (1998). Laxative-induced hypokalaemia. Ann Emerg Med.

[27] Hawkins RC (2003). Gender and age as risk factors for hypokalemia and hyperkalemia in a Asian population. Clin Chim Acta.

[28] Lindner G, Pfortmüller CA, Leichtle AB (2014). Age-related variety in electrolyte levels and prevalence of dysnatremias and dyskalemias in patients presenting to the emergency department. Gerontology.

[29] Edmonds CJ, Jasani BM, Smith T (1975). Total body potassium and body fat estimation in relationship to height, sex, age, malnutrition and obesity. Clin Sci Mol Med.

[30] Yoon HE, Choin BS (2014). The renin–angiotensin system and aging in the kidney. Korean J Intern Med.

